# Supplementary material for: Hypoxia-Targeted Immunotherapy with PD-1 Blockade in Head and Neck Cancer
Source: Cancers (Basel). 2024 Aug 29;16(17):3013. doi: 10.3390/cancers16173013 (PMC11394489; doi:10.3390/cancers16173013)
Supplement: Supplementary file 1 [file cancers-16-03013-s001.zip › cancers-3158181-supplementary.pdf]

Figure S1

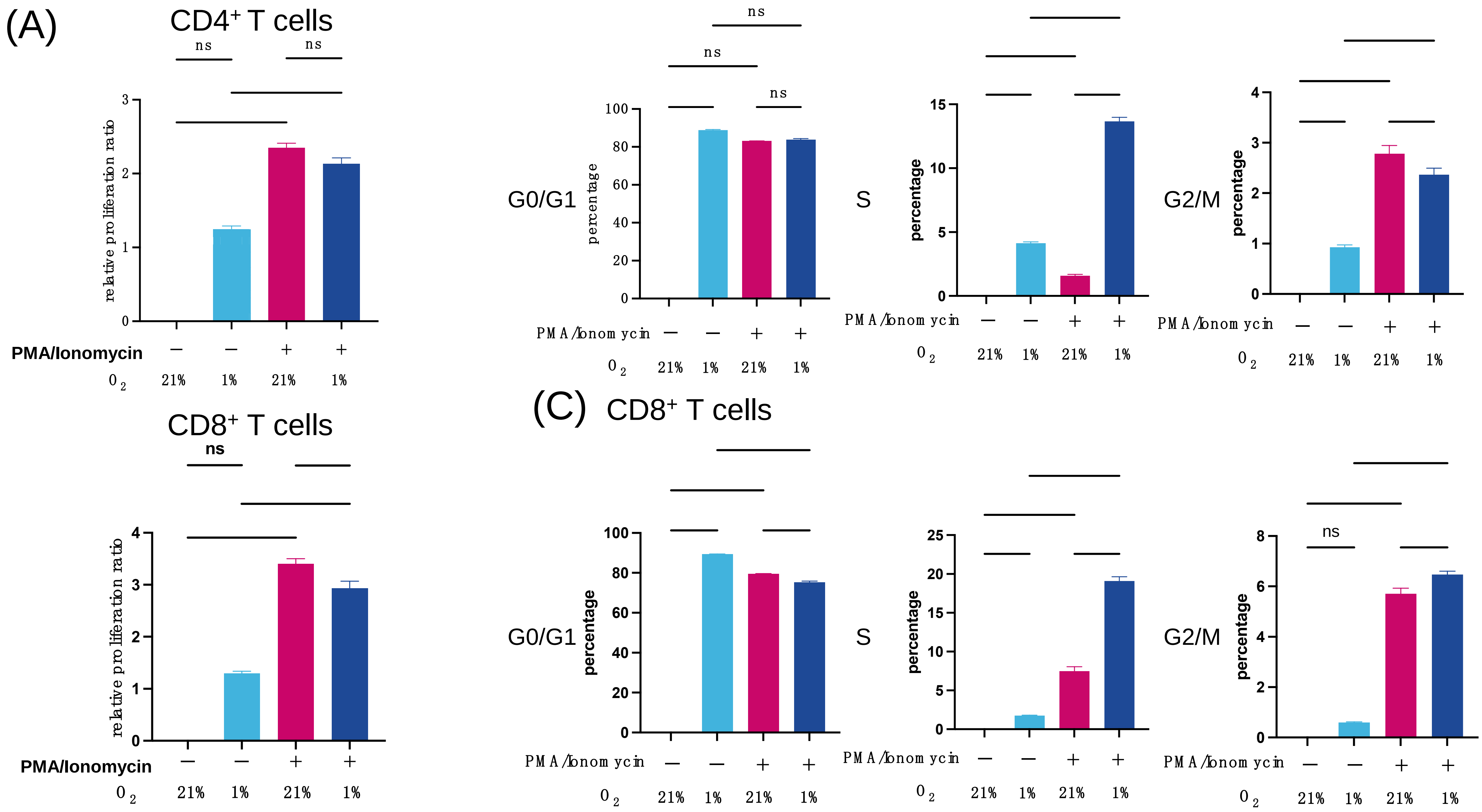
(A)
CD4+ T cells
ns
ns
relative proliferation ratio
0
1
2
3
PMA/Ionomycin
O2
21%
1%
21%
1%
CD8+ T cells
ns
relative proliferation ratio
0
1
2
3
4
(B) CD4+ T cells
G0/G1
S
G2/M
percentage
ns
(C) CD8+ T cells
G0/G1
S
G2/M
percentage
ns

Figure S1.Viability and proliferation of immune cells under normoxic conditions.

(A) Cell proliferation ratio of CD4+ and CD8+ T cells were assessed using the CCK-8 assay under normoxic (21%O2) or hypoxic (1%O2) conditions with or without PMA/Ionomycin stimulation for 48 h. Experiments were performed in triplicate. Bars and error bars represent the mean and SD, respectively ($^*p < .05$, $^{**}p < .01$, $^{***}p < .001$, $^{****}p < .0001$, one-way ANOVA).
(B, C) Cell cycle analysis of CD4+ T cell and CD8+ T cell by propidium iodide staining and flow cytometry under normoxic or hypoxic conditions, with or without PMA/Ionomycin stimulation for 24 h. Experiments were performed in triplicate. Bars and error bars represent the mean and SD, respectively ($^*p < .05$, $^{**}p < .01$, $^{***}p < .001$, $^{****}p < .0001$, one-way ANOVA).

Figure S2

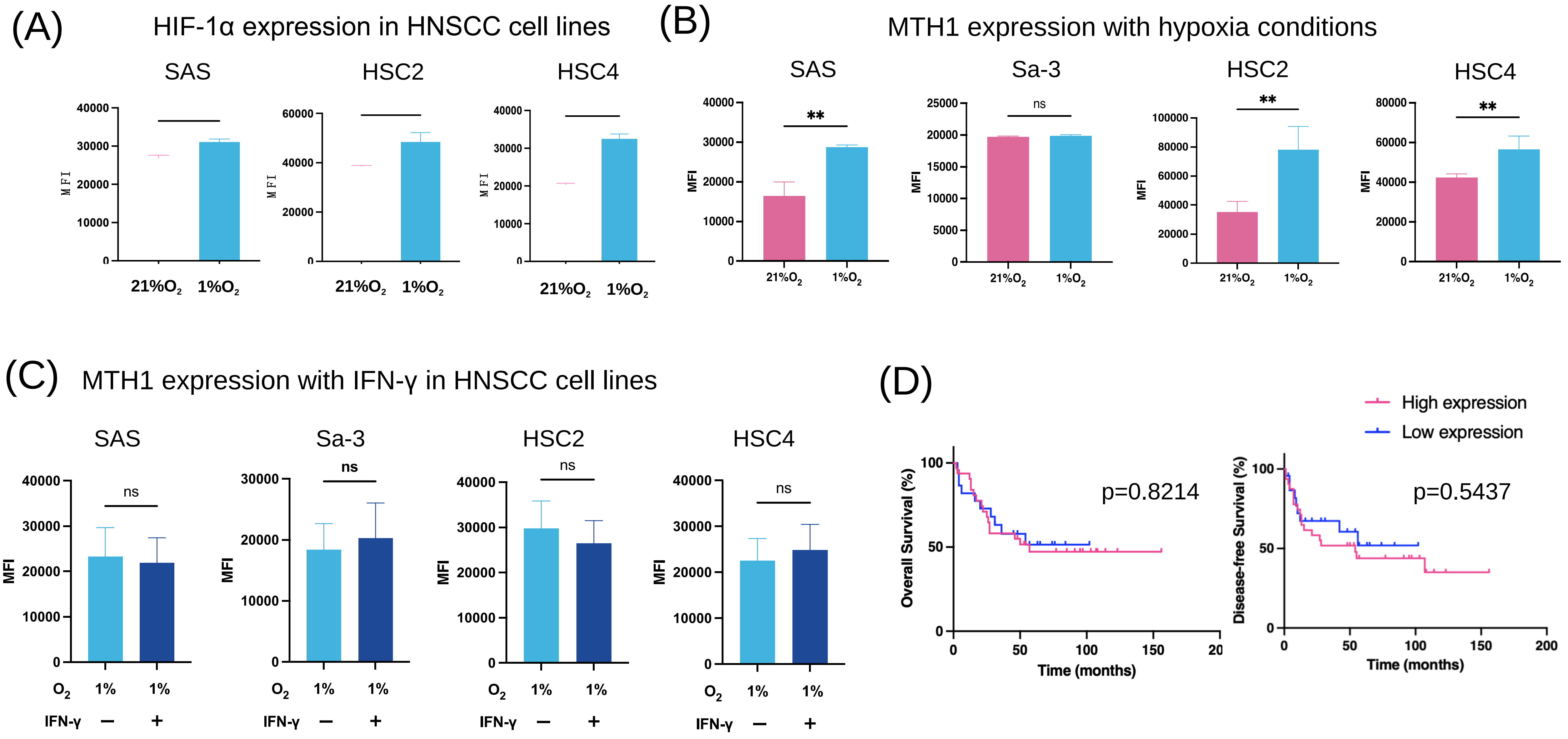

(A)
HIF-1α expression in HNSCC cell lines
SAS
HSC2
HSC4
MFI
21%O2
1%O2
(B)
MTH1 expression with hypoxia conditions
SAS
Sa-3
HSC2
HSC4
MFI
**
ns
21%O2
1%O2
(C)
MTH1 expression with IFN-γ in HNSCC cell lines
SAS
Sa-3
HSC2
HSC4
MFI
ns
O2
1%
IFN-γ
(D)
High expression
Low expression
Overall Survival (%)
p=0.8214
Disease-free Survival (%)
p=0.5437
Time (months)

Figure S2. Hypoxia-related protein expression in HNSCC.

(A) Average values of mean fluorescence intensity (MFI) of intracellular staining analysis of HIF-1α expression in HNSCC cell lines. Secondary antibody with isotype control was used as a control.

(B) Averages values of mean fluorescence intensity (MFI) of MTH1 expression in hypoxic conditions. Bars and error bars represent the mean and SD, respectively (*p < .05, **p < .01, ***p < .001, ****p < .0001, Student's t test). Experiments were performed in triplicate. Bars and error bars represent the mean and SD, respectively. (*p < .05, **p < .01, ***p < .001, ****p < .0001, Student's t test)

(C) Average values of mean fluorescence intensity (MFI) of intracellular staining analysis of MTH1 expression in HNSCC cell lines with or without IFN-γ stimulation. The experiments were performed in triplicate. Bars and error bars represent the mean and SD, respectively (*p < .05, **p < .01, ***p < .001, ****p < .0001, Student's t test).

(D) Overall survival and disease-free survival rates according to the intensity of MTH1 expression in patients with oropharyngeal carcinoma. IHC scores ≥ 5 were defined as high expression, and scores < 5 were defined as low expression.

Figure S3

(A)

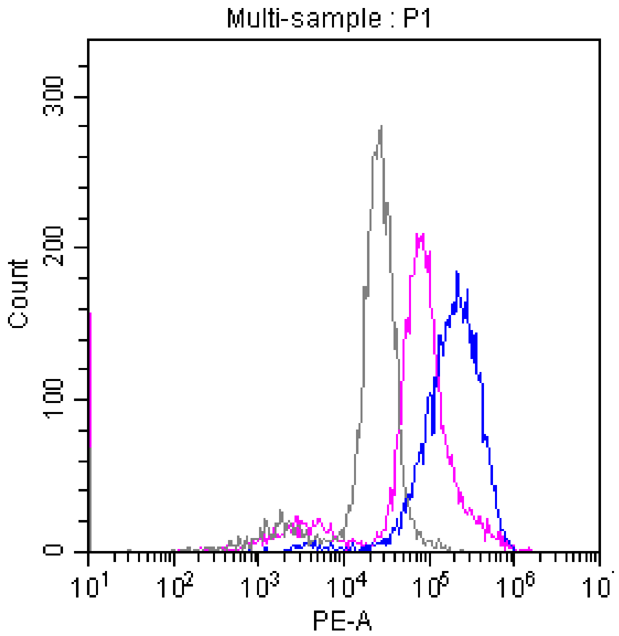

(B)

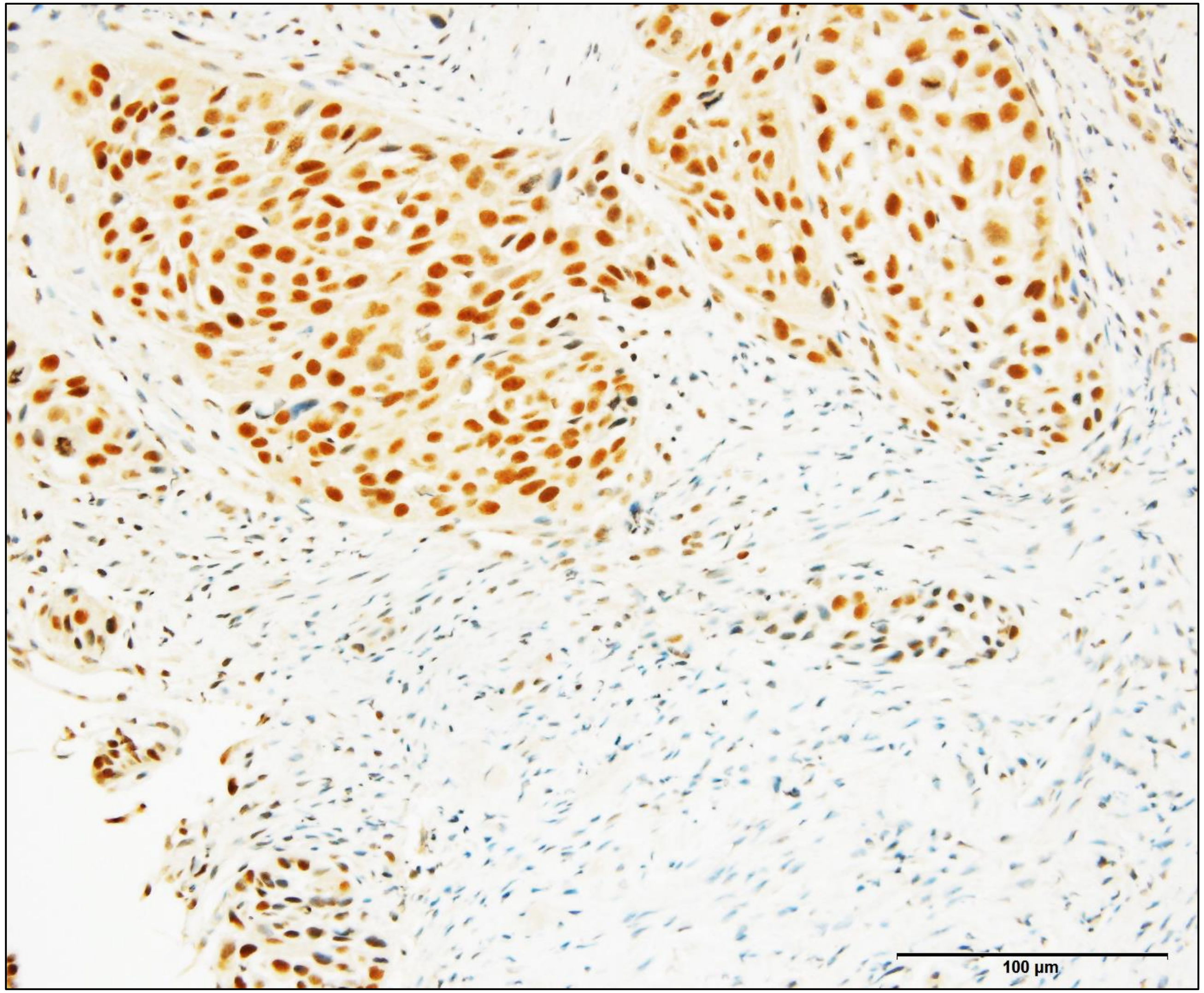

(C)

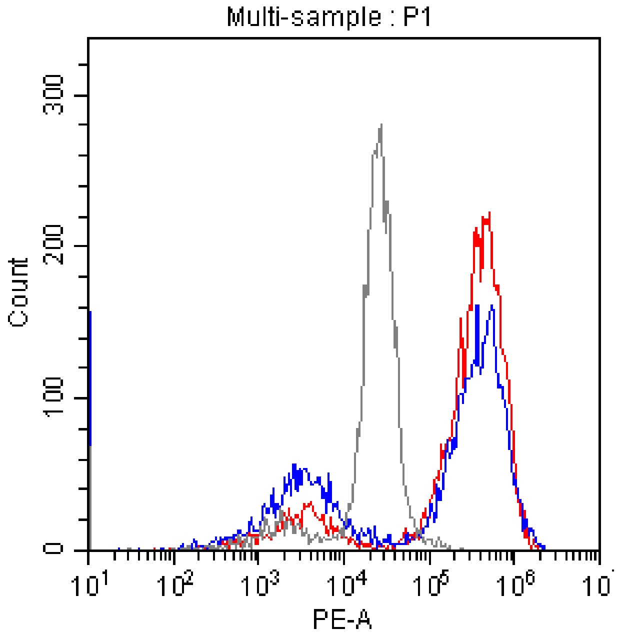

Figure S3. Tumoral expression of MTH1.

(A) The expression levels of MTH1 in normal human bronchial epithelial cells (NHBE) and SAS were evaluated by Flow cytometry. Black, isotype control; pink, NHBE; blue, SAS.

(B) The representative image of immunohistochemical staining of MTH1 in head and neck squamous cell cancer.

(C) The expression levels of MTH1 in normal human bronchial epithelial cells (NHBE) with hypoxic conditions. Black, isotype control; blue, 21%O_2; red, 1%O_2.
